# Retrospective Analysis of the Pharmaco-Utilization of VEGF Inhibitors and Health Care Costs among Patients with Wet Age-Related Macular Degeneration and Other Ocular Diseases in Italy

**DOI:** 10.3390/ijerph19052548

**Published:** 2022-02-23

**Authors:** Valentina Perrone, Melania Dovizio, Chiara Veronesi, Rita Citraro, Adele De Francesco, Stefania Dell’Orco, Gianluca Di Manno, Arrigo Paciello, Anna Maria Resta, Fabrizio Quarta, Nicola Ferrante, Daniela Ritrovato, Luca Degli Esposti

**Affiliations:** 1 CliCon S.r.l. Società Benefit Health, Economics & Outcomes Research, 40121 Bologna, Italy; melania.dovizio@clicon.it (M.D.); chiara.veronesi@clicon.it (C.V.); luca.degliesposti@clicon.it (L.D.E.); 2 Unita’ Operativa di Farmacologia Clinica e Farmacovigilanza, Azienda Ospedaliero-Universitaria “Mater Domini”, Università Magna Grecia di Catanzaro, 88100 Catanzaro, Italy; citraro@unicz.it (R.C.); defrancescoad@gmail.com (A.D.F.); 3 Azienda Sanitaria Locale (ASL) Roma 6, Albano Laziale, 00100 Rome, Italy; stefania.dellorco@aslroma6.it (S.D.); gianluca.dimanno@aslroma6.it (G.D.M.); 4 Agenzia di Tutela della Salute (ATS) Bergamo, 24100 Bergamo, Italy; arrigo.paciello@ats-bg.it; 5 Struttura Complessa di Farmacia Territoriale Area Vasta 1, 61032 Fano, Italy; anna.resta@sanita.marche.it; 6 U.O. Epidemiologia e Statistica, Azienda Sanitaria Locale (ASL) Lecce, 73100 Lecce, Italy; uose@ausl.le.it; 7 Novartis Farma S.p.A., Origgio, 21100 Varese, Italy; nicola.ferrante@novartis.com (N.F.); daniela.ritrovato@novartis.com (D.R.)

**Keywords:** age-related macular degeneration, anti-VEGF, real-world evidence, pharmaco-utilization, health care costs

## Abstract

This Italian retrospective study aimed to analyze the pharmaco-utilization of anti-VEGF drugs and health care costs among patients with wet age-related macular degeneration (wAMD) or other ocular diseases. A retrospective analysis was performed on administrative databases of Italian entities covering approximately six million individuals. Across January 2010–December 2017, patients aged ≥50 years with a prescription of intravitreal anti-VEGFs were included as “wAMD” patients [by wAMD hospitalization or intravitreal injections] or as “other ocular diseases” patients [by hospitalization for other ocular disorders or intravitreal injections, with concomitant diabetes diagnosis or dexamethasone treatment]. The date of first matching of inclusion criteria was index-date. wAMD-cohort. Overall, 3879 patients were included; at index-date, 82.2% were treated with Ranibizumab, 15.8% with Aflibercept, and 2% with Pegaptanib. During the follow-up, the mean/annual anti-VEGF prescription [3.6 (first-year)–0.8 (third-year)] and the total expenditure [5799.84 € (first-year)–3212.84 € (third-year)] decreased. Other ocular diseases-cohort. Overall, 2646 patients were enclosed; 85.9% were treated with Ranibizumab, 13.5% with Aflibercept, and 0.6% with Pegaptanib. During the follow-up, the mean/annual anti-VEGF prescription [3.3 (first-year)–0.5 (third-year)] and the total cost [7196.83 € (first-year)–5162.68 € (third-year)] decreased. This observational study highlighted a decline in anti-VEGF prescriptions over time in both cohorts, suggesting a trend of under-treatment that could worsen the patients’ clinical outcomes and increase health care resource consumption.

## 1. Introduction

Age-related macular degeneration (AMD) is a degenerative retinal disease that causes progressive loss of central vision [1]. Neovascular AMD (also known as wet AMD, wAMD) is an advanced form of AMD characterized by choroidal neovascularization, which results in vision-impairing exudation and hemorrhage, and affects the macula, and the photoreceptor dense area in the central portion of the retina that is responsible for central, high-resolution vision [1,2]. Elevated vascular endothelial growth factor (VEGF) levels have been shown to be implicated in the pathogenesis of wAMD [3,4]. In addition, high levels of VEGF are also responsible for pathological neovascularization in diabetic retinopathy (DR), comprising the proliferative diabetic retinopathy (PDR) and the diabetic macular edema (DME), and in other ocular diseases (i.e., retinal vein occlusion (RVO), myopic choroidal neovascularization (mCNV)) [4].

Several epidemiological studies showed that the prevalence of AMD (both wet and dry AMD) ranged between 0.2% and 5.6% in Europe, with a value for Italy of 2.1% [5]. Worldwide estimates approximated that 30–50 million people are affected by AMD, and these numbers are expected to increase over time because of the aging population [5,6]. Up to now, no cure or treatments have been shown to definitely restore the vision already lost in wAMD patients [1,2]. Due to the recognition of the pathogenetic role of VEGF in ocular vascular diseases (including wAMD), the use of VEGF-specific antagonists (anti-VEGF) represents the current therapy [7], which demonstrated high rates of success in the clinical practice [7].

Currently in Italy, the anti-VEGF medicines for intravitreal use are: Aflibercept, Bevacizumab, Brolucizumab, and Ranibizumab [8]. Pegaptanib was used until 2018 when it was no longer marketed following the withdrawal decision by the European Commission [8]. Aflibercept and Ranibizumab are indicated for the treatment of wAMD and of other ocular diseases such as DME, mCNV, and RVO [8,9]; Brolucizumab and Pegaptanib for wAMD, while Bevacizumab represents an off-label therapeutic option for wAMD [8,9].

In the registration trials for Ranibizumab and Aflibercept, after the first year of treatment, monthly (treatment every four weeks) and bimonthly (treatment every eight weeks after 3-month loading) fixed regimens were used, respectively [10].

In addition to harming patients’ lives, wAMD and other ocular disorders cause significant adverse consequences for the economy due to the impairment of patient quality of life. Currently, limited data on the use of anti-VEGF therapy for wAMD and other ocular diseases patients in Italian routine practice are available. Thus, the objectives of the present study were to analyze the treatment pattern, the pharmaco-utilization of anti-VEGF drugs, and health care costs among patients affected by wAMD or other ocular diseases in a clinical practice Italian setting.

## 2. Materials and Methods

### 2.1. Data Source

The study was carried out using data extracted from the administrative databases from a pool of Italian health care entities from Marche, Lombardy, Calabria, Apulia, and Lazio Regions, which covered approximately six million inhabitants.

The administrative data related to health care services and financial and clinical information are routinely and continuously collected in large databases. The Italian National Health Service (NHS) provides health care to all inhabitants. Health care is publicly financed, and services are either free or involve co-payment [11]. The Italian NHS is decentralized and organized at three levels: national, regional, and local. Data on the services provided to residents are collected by hospitals and local health care structures [11].

Within the administrative flows, the anonymous univocal numeric code assigned to each patient allowed for the electronic linkage of all records for each subject across the databases. Specifically, data-linkage was performed among the following databases: demographic database (to collect data on patients’ demographic characteristics), pharmaceutical database (to collect data on prescription of drugs reimbursed by the Italian NHS, such as Anatomical-Therapeutic Chemical (ATC) code, and prescription date), hospitalization database (to obtain information on discharge diagnoses at any level classified according to the International Classification of Diseases, Ninth Revision, Clinical Modification (ICD-9-CM) and date of diagnoses), and diagnostic tests and specialist visits database (containing the date of prescription, type, description activity of diagnostic tests, and procedure for patients in analysis).

The anonymous univocal numeric code ensured total compliance with the European General Data Protection Regulation (GDPR) (2016/679). No identifiers related to patients were provided to the authors. All the results of the analyses were produced as aggregated summaries, and the data could not be assigned, either directly or indirectly, to a single institution, department, doctor, individual, or individual prescribing behaviors. Based on the Data Privacy Guarantor Authority (General Authorization for personal data treatment for scientific research purposes—n.9/2014), informed consent was not required, as its collection would be impossible for organizational reasons. According to the Italian law on the conduction of observational analyses, the ethics committee of each participating entity was notified and approved of the study (the details are reported in the Institutional Review Board Statement below).

### 2.2. Study Design and Study Population

This was an observational retrospective cohort study in which all subjects aged ≥50 years with at least one prescription of intravitreal anti-VEGF drugs, between 1 January 2010 to 31 December 2017 were screened for eligibility. Patients were considered as “wAMD patients” (wAMD-cohort) if they presented: (i) at least one hospitalization with a primary or secondary discharge diagnosis of wAMD (ICD-9-CM code: 362.52) or (ii) outpatient services (intravitreal injection of therapeutic substances, ICD-9-CM code 14.75). The exclusion criteria were: (i) the presence of at least intravitreal injection of therapeutic substances (ICD-9-CM code 14.75) with a concomitant diagnosis of diabetes [detected through the presence of at least one hospitalization diagnosed with diabetes (ICD-9-CM code 250) or at least two prescriptions of antidiabetic drugs (ATC code A10)] or (ii) a concomitant treatment with dexamethasone (ATC code S01BA01) or (iii) a diagnosis of other ocular diseases such as RVO (ICD-9-CM code: 362.3), mCNV (ICD-9-CM code: 362.16), DME (ICD-9-CM code: 362.07), PDR (ICD-9-CM code: 362.02), or DR (ICD-9-CM code: 362.0-excluded codes: 362.02 e 362.07).

Patients were considered as affected by other ocular diseases (other ocular diseases-cohort) if they presented: (i) at least one hospitalization with a primary or secondary discharge diagnosis of ocular disorders indicated for treatment with anti-VEGF drugs (such as DME, RVO, PDR, DR, or mCNV) or (ii) outpatient services (intravitreal injection of therapeutic substances, ICD-9-CM code 14.75, with the concomitant diagnosis of diabetes or treatment with dexamethasone (ATC code S01BA01)). Patients with at least one hospitalization for wAMD (ICD-9-CM code: 362.52) in the period of inclusion were excluded from the other ocular diseases-cohort.

The two cohorts were analyzed separately. Within the inclusion period, the date that corresponded to the first prescription of an anti-VEGF drug or to hospitalization for wAMD or other ocular disorders (for patients without anti-VEGF prescription) was the index-date.

### 2.3. Study Variable

For all patients included in the study, at the index-date and during the follow-back period (12 months before the index date), the age, gender, and the therapeutic treatments have been recorded. During the all available follow-up (at least 12 months) the occurrence of death, and the presence of complications [in terms of mechanical complication due to corneal graft (code 996.51), mechanical complication of intraocular lens (code 996.53), infection and inflammatory reaction due to ocular lens prosthesis and prosthetic orbital implant (code 996.69), postoperative infection, and complications (codes 998.5, 998.8, 998.9), infections and sepsis (code 999.3)] were evaluated. During the follow-up period (up to three years after the index date), the mean annual number of anti-VEGF drug prescriptions and the direct health care costs were gathered for all patients.

### 2.4. Treatment Patterns, Pharmaco-Utilization, and Cost Analyses

In all patients treated with anti-VEGF drugs, the use of these medications at the index date was evaluated using the following ATC codes: (i) Aflibercept (ATC code S01LA05), (ii) Pegaptanib (ATC code S01LA03), and (iii) Ranibizumab (ATC code S01LA04). The pharmaco-utilization of the anti-VEGF agents was evaluated in terms of the mean annual number of prescriptions per patient during the first, second, and third year of follow-up. In addition, the total direct costs related to drug prescriptions, hospitalizations, and outpatient specialist service prescriptions were assessed in all alive patients during the first, second, and third year of follow-up. The health care cost analysis was conducted from the perspective of Italian NHS. The costs were reported in euros (€) and using the Italian NHS purchase price. Hospitalization costs were determined using the DRG (diagnosis-related groups) tariffs. The cost of instrumental and laboratory tests was defined according to the tariffs applied by the region.

### 2.5. Statistical Methods

Continuous data were summarized in terms of the number of observations, mean, and standard deviation (SD). Categorical data were summarized in terms of the number of patients providing data. The frequency counts and percentages of patients in each category were provided. Percentages were calculated using the number of observations with non-missing values as the denominator. An ANOVA test was performed to compare means across one or more variables of repeated observations; patients with missing values were excluded from the analysis. Statistical significance was accepted at *p* < 0.05. All statistical analyses were performed using STATA SE, version 17.0(StataCorp LP, College Station, TX, USA). According to “Opinion 05/2014 on Anonymization Techniques” drafted by the “European Commission Article 29 Working Party”, the analyses involving fewer than three patients were not reported as they were potentially traceable to single individuals. Therefore, results referred to ≤3 patients were reported as NI (not issuable).

## 3. Results

### 3.1. Patients from the wAMD-Cohort

Demographic and clinical characteristics of wAMD patients are reported in Table 1. Based on the inclusion criteria, 3879 wAMD patients were included with a mean age of 75.3 years (43.5% male).

**Figure 1:**
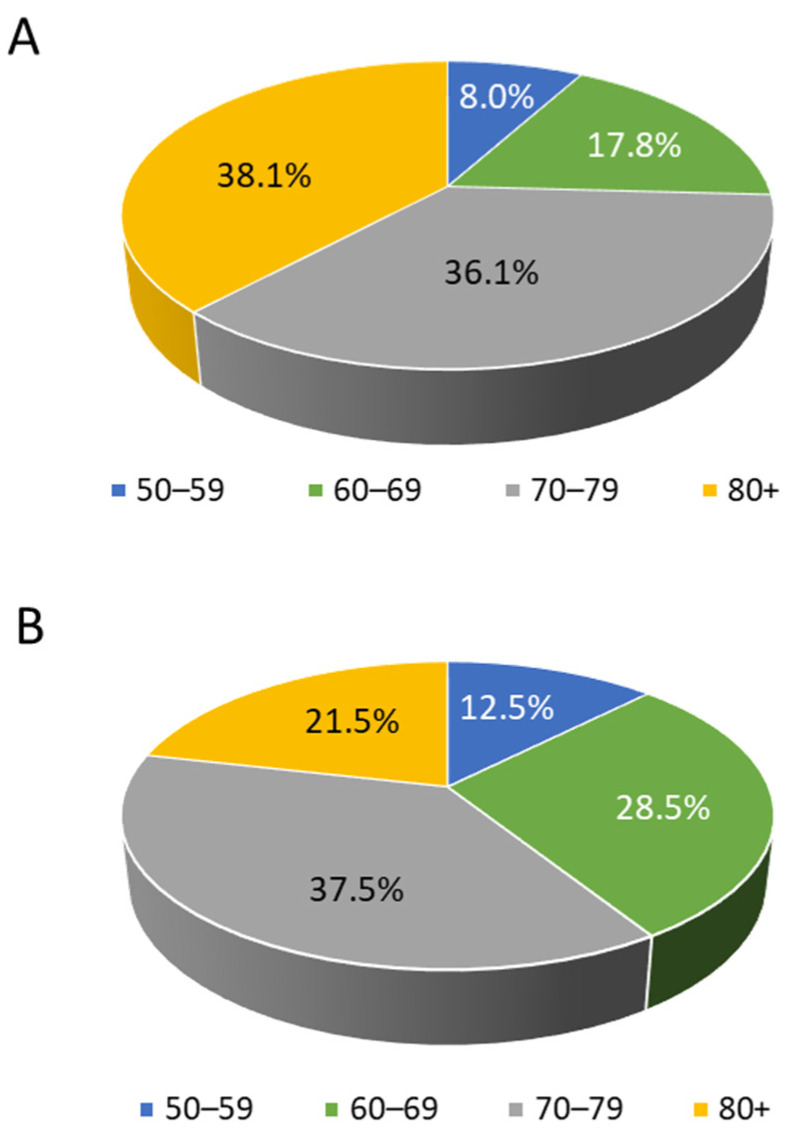
Age distribution among wAMD patients (**A**) and those affected by other ocular diseases (**B**).

Patients were stratified based on age range: as shown in Figure 1A, 8% of patients ranged 50–59 years, 17.8% went 60–69 years, 36.1% ranged 70–79 years, and 38.1% were more than 80 years old. The mean observational period for all patients was around 4.0 years (Table 1). A percentage of 13.3% of wAMD patients died during the follow-up period (Table 1), and 20.1% presented complications (Table 1).

The analysis of pharmaco-utilization of three anti-VEGF molecules has shown that at the index date, 82.2% of wAMD patients were under Ranibizumab treatment, 15.8% were treated with Aflibercept, and 2% with Pegaptanib as monotherapy (Table 2).

**Figure 2:**
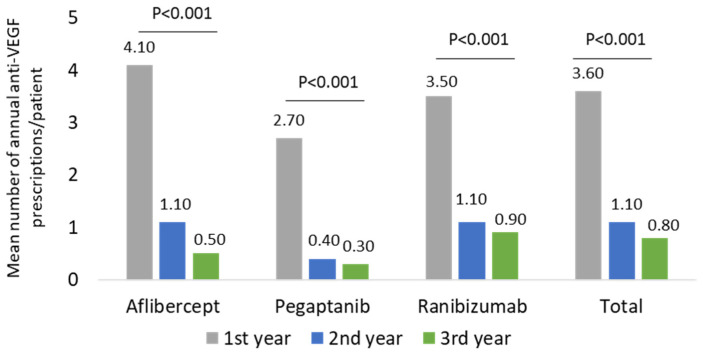
Mean number of annual anti-VEGF prescriptions/patient during the follow-up among the wAMD-cohort.

For all three anti-VEGF molecules, the mean annual number of prescriptions per patient was evaluated during the entire follow-up period and at the first, second, or third year. As reported in Figure 2 and in Supplementary Table S1, the mean annual number of prescriptions for all drugs significantly (*p* < 0.001) decreased during the follow-up period: the overall value ranged 3.6 ± 2.0 during the first year, 1.1 ± 1.8 in the second year, and 0.8 ± 1.7 during the third year.

Among wAMD patients stratified by age ranges, for all three anti-VEGF molecules, the mean annual number of prescriptions per patient was evaluated during the entire follow-up period and at the first, second, or third year. As reported in Table 3, in all age groups, the mean annual number of prescriptions for all drugs significantly (*p* < 0.001) decreased during the three year follow-up period (in 50–59 years, 4.3 to 0.6; in 60–69 years, 3.6 to 0.9; in 70–79 years 3.8 to 1.0; more than 79 years, 3.6 to 0.6). The same scenario was found among patients affected by other ocular diseases (not shown).

**Figure 3:**
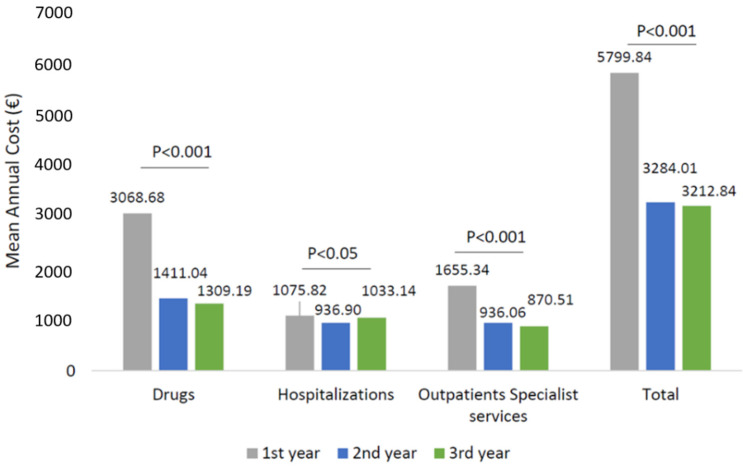
Health care-related costs during the follow-up among patients in the wAMD-cohort.

**Figure 4:**
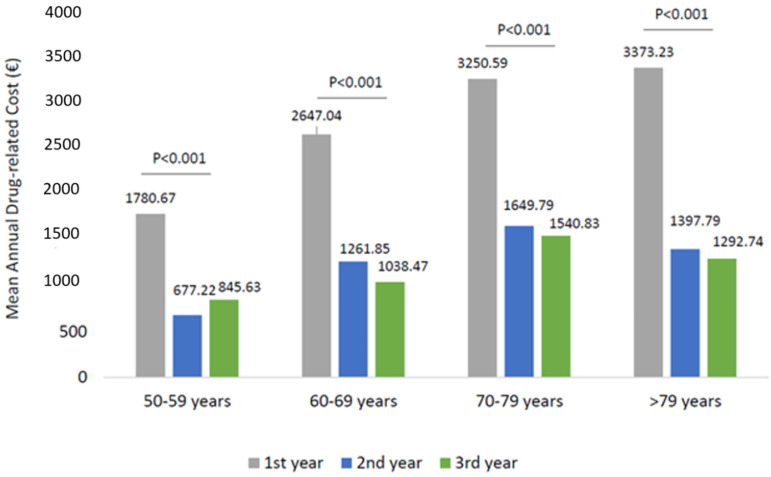
Drug-related costs during the follow-up among patients of wAMD-patients stratified by age classes.

The analysis of the health care-related costs has shown that in wAMD patients, the mean total expenditure during the first year of follow-up was around 5799.84 € and decreased significantly to 3284.01 and 3212.84 € during the second and third year of follow-up, respectively (Figure 3). The trend of total cost reduction observed during the three years of follow-up was mainly due to a reduction (*p* < 0.001) in drug-related costs during the second and third year (Figure 3), with a minor impact of the hospitalization-related costs and outpatient specialist service costs. Among the wAMD patients stratified by age classes, a significant reduction in drug-related costs alongside the follow-up was found in all age subgroups (Figure 4). In particular, the most prominent effect was evidenced in patients older than 70 years (Figure 4).

### 3.2. Patients from the Other Ocular Disease-Cohort

Demographic and clinical characteristics of patients are reported in Table 1. Based on the inclusion criteria, 2646 patients affected by other ocular diseases were enclosed; the mean age was 71.4 years, and 54.9% were male (Table 1). The stratification of other ocular disease-cohort patients showed that 12.5% of them ranged from 50 to 59 years, 28.5% were 60–69 years, 37.5% were 70–79 years, and 21.5% were more than 80 years old (Figure 1B). The mean observational period of patients was around 4.0 years (Table 1); 18.4% of them died during the follow-up period (Table 1) and 27.9% presented complications (Table 1).

The analysis of treatment patterns in these patients highlighted that, at the index date, 85.9% of patients were under Ranibizumab treatment, 13.5% were treated with Aflibercept, and 0.6% with Pegaptanib (Table 2).

**Figure 5:**
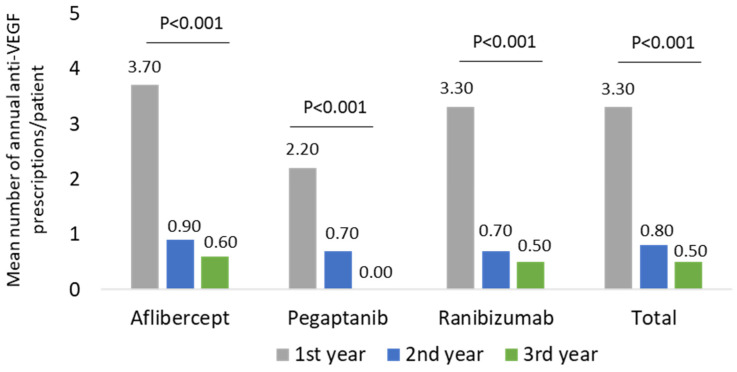
Mean number of annual anti-VEGF prescriptions/patient during the follow-up among other ocular disease-cohort patients.

As reported in Figure 5 and Supplementary Table S2, in patients belonging to other ocular disease-cohorts, the mean annual number of prescriptions for all anti-VEGF drugs significantly (*p* < 0.001) decreased during the follow-up period: the overall value ranged 3.3 ± 1.9 during the first year, 0.8 ± 1.5 in the second year, and 0.5 ± 1.3 during the third year (Figure 5 and Supplementary Table S2).

**Figure 6:**
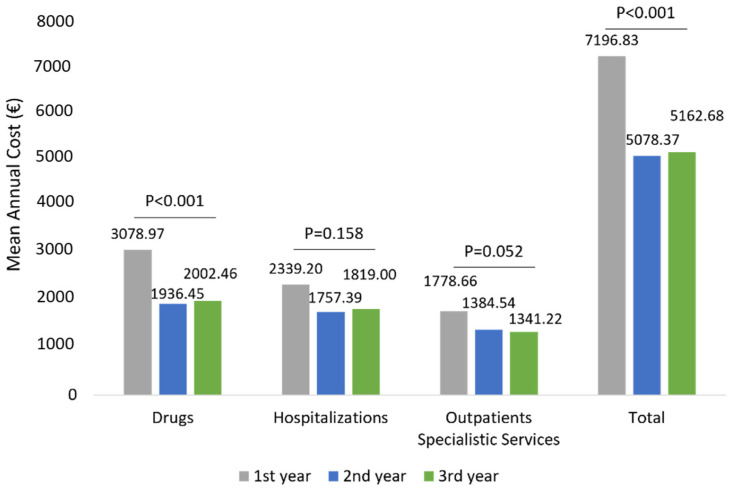
Health care-related costs during the follow-up among patients of other ocular disease-cohorts.

**Figure 7:**
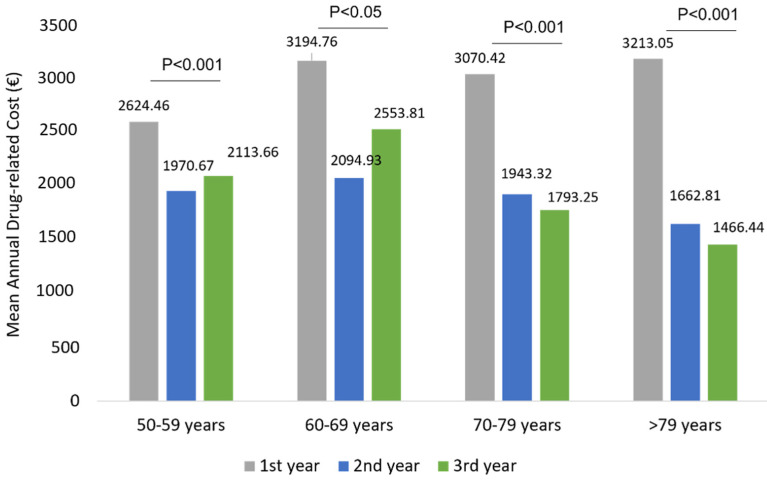
Drug-related costs during the follow-up among patients of other ocular disease patients stratified by age classes.

The mean total health care-related costs was around 7196.83 € during the first year of follow-up and significantly (*p* < 0.001) decreased to 5078.37 € and 5162.68 € during the second and third year of follow-up, respectively (Figure 6). Similarly to wAMD patients, in patients affected by other ocular diseases, the total cost reduction observed during the three years of follow-up was mainly due to a statistically significant reduction in drug-related costs (*p* < 0.001) during the second and third year (Figure 6), while hospitalization-related costs and those of outpatient specialistic services remained nearly the same over the entire follow-up period. Among patients with other ocular diseases stratified by age classes, a significant reduction in drug-related costs alongside the follow-up was found in all age subgroups (Figure 7). Similarly to the wAMD cohort, the most prominent effect was seen in patients older than 70 years (Figure 7).

## 4. Discussion

In this observational retrospective study, we provided a thorough investigation of anti-VEGF drug utilization and health care-related costs during 2010–2017 among patients with wAMD or other ocular diseases followed up to three years, in an Italian setting of clinical practice. The pharmaco-utilization analysis showed that the distribution of patients based on the two most prescribed anti-VEGF agents, Ranibizumab and Afilibercept, was unbalanced toward Ranibizumab (more than 80% of patients have been prescribed this drug). These results are in line with retrospective analyses among Italian patients affected by macular degeneration [12,13] and could be explained by the fact that Ranibizumab obtained the first reimbursement in 2008, with Aflibercept in 2014. Although a recent analysis on the use of anti-VEGF agents among Italian wAMD patients conducted by AIFA showed that almost half of treatments (44.6%) were with Ranibizumab, followed by Aflibercept (33.1%), but starting from 2016 to 2019, Aflibercept showed an increasing trend with the anti-VEGF having the highest number of eye treatments/month, while Ranibizumab use remained constant [8,14].

In the present study, a sub-optimal prescribing appropriateness of all anti-VEGF drugs was found since a downward trend in the mean number of prescriptions up to three years of follow-up was reported for both the two cohorts. During the first year of follow-up, the number of anti-VEGF prescriptions/patients averaged 3.6 (with Aflibercept being 4.1, Ranibizumab and Pegaptanib reaching 3.5 and 2.7, respectively). These data were confirmed by the OSMED 2019 Report released by AIFA, which stated 3.6 mean annual injections during the first year of follow-up (3.8 for Aflibercept, 2.6 for Pegaptanib, and 3.5 for Ranibizumab) [14]. Otherwise, our results showed that among the wAMD or other ocular disease patients during the second and third year of follow-up, anti-VEGF drugs were prescribed almost as a mono-administration per year. This undertreatment phenomenon has also been widely reported [15] in retrospective studies among Italian patients with macular degeneration [12,16]. The factors responsible for anti-VEGF undertreatment could be related to patients, clinic organizational structure, and physicians [15]. From the patient’s perspective, the psychosocial burden and the economic burden associated with treatments and monitoring visits in terms of care and travel expenses for both patients and caregivers, significantly impacted treatment adherence [17,18]. At the same time, the therapeutic management of wAMD patients and those with other ocular diseases requires monitoring visits, which are the most time and resource-consuming items of patient care, making monthly regimens unachievable in clinical practice [15,19,20]. In addition, 47% of therapy discontinuation cases have been accountable from clinical decisions based on the stability of the disease and other characteristics/comorbidities of the patients [12,15]. On the whole, in long-term and clinical routine settings, significant gaps and defiance, especially in the treatment and management of wAMD patients, pose barriers to achieving optimal clinical outcomes [21]. Many factors may contribute to additional challenges, leading to suboptimal long-term outcomes among these patients such as delays in treatment approval and initiation, lapses in physician regimentation of anti-VEGF injection, and inadequate patient adherence to treatment and monitoring [21]. Although we observed a health care cost reduction, presumably related to the decrease in anti-VEGF prescriptions, our analysis, due to the limited follow-up, did not consider the clinical and economic consequences of undertreated patients. The analysis of anti-VEGF prescriptions and drug-related expenses in patients stratified by age classes showed that older patients (more than 70 years) are likely to be prescribed with more anti-VEGF prescriptions, and their management was associated with higher drug costs during the first year of follow-up. However, in these older patients, with the progression of the years, the prescriptions and drug cost significantly decreased with more impact, with respect to the younger population.

The sub-optimal therapeutic management of patients with macular degeneration, especially in older patients, may result in a negative outcome such as complete visual loss with the consequent increase in economic burden for these disabled persons, their caregivers, and society [22]. In the EAGLE study, routine clinical practice management of patients with AMD among the Italian population was reported; in patients, the anti-VEGF undertreatment was observed, and the authors stated that more injections and the amelioration of treatment regimen correlated with better visual outcomes [23].

The limitations of the present study were derived by using anonymized data from administrative databases. Although region/local health unit administrative databases have progressively improved the data quality, some information may have been missed. Another limitation is that the quality of life and the visual outcomes in the sample population were not monitored because of the limited time horizon available and the data source (administrative databases) used, which did not allow to retrieve such information.

However, although experimental evidence (despite limited) is available in the literature on the real-world data of anti-VEGF pharmaco utilization and health care resource consumption in Italy, the strengths of the current study deserve to be reported. This retrospective analysis was carried out by considering a quite extended study period (from 2010–2017) embracing more recent years. In addition, it was a multicentric analysis over five regions geographically distributed across Italy. All patients were followed-up with for a considerable period (up to three years) after the initiation of anti-VEGF treatment and correlations between the number of prescriptions and the total direct costs were performed among the same study population. Moreover, these results could support health policy decision-making, especially among the Italian National Social Welfare Institute, to manage the higher costs related to benefits for disabilities derived from the inadequate management of patients affected by ocular diseases.

## 5. Conclusions

In conclusion, in the present study, an overview of the VEGF inhibitor utilization and health care related costs among wAMD patients and those affected by other ocular diseases was performed in Italian settings of clinical practice. The prescriptions of VEGF inhibitors among these patients decreased over time. This phenomenon could be related to visual stabilization, but also to a trend of undertreatment that could ultimately lead to the worsening of clinical outcomes with the amplification of the overall economic burden of patients with ocular disorders.

## Figures and Tables

**Table 1:** Demographic and clinical characteristics of wAMD patients and those affected by other ocular diseases.

	wAMD-Cohort	Other Ocular Diseases-Cohort
N	3879	2646
Age (mean ± SD)	75.3 ± 9.7	71.4 ± 9.5
Male (n, %)	1688 (43.5)	1452 (54.9)
Observation period (years) (mean ± SD)	4.1 ± 2.0	4.0 ± 2.0
Death (n, %)	515 (13.3)	486 (18.4)
Presence of complications (death included) (n, %)	780 (20.1)	738 (27.9)

**Table 2:** Use of anti-VEGF drugs at index date among wAMD patients and those affected by other ocular diseases.

	wAMD-Cohort	Other Ocular Diseases-Cohort
N of treated patients	3387	1912
Aflibercept, n (%)	536 (15.8)	259 (13.5)
Pegaptanib, n (%)	67 (2.0)	11 (0.6)
Ranibizumab, n (%)	2784 (82.2)	1642 (85.9)

**Table 3:** Mean number of annual anti-VEGF prescriptions/patient during the follow-up among the wAMD-cohort stratified by age ranges.

	All Available Fw-Up, Mean (sd)	1st Year Fw-Up, Mean (sd)	2nd Year Fw-Up, Mean (sd)	3rd Year Fw-Up, Mean (sd)	*p*-Value
Aflibercept					
50–59 years	4.6 (3.5)	3.3 (1.6)	1.3 (1.9)	1.8 (1.3)	0.090
60–69 years	5.9 (4.1)	4.1 (2.0)	1.6 (2.0)	0.5 (1.2)	<0.001
70–79 years	5.8 (3.5)	4.3 (1.9)	1.2 (1.8)	0.7 (1.4)	<0.001
>79 years	5.3 (3.3)	4.0 (1.7)	0.9 (1.5)	0.3 (1.1)	<0.001
Pegaptanib					
50–59 years	-	-	-	-	-
60–69 years	NI	2.8 (1.7)	NI	NI	-
70–79 years	6.1 (5.3)	3.1 (1.8)	0.8 (1.7)	0.6 (1.2)	<0.001
>79 years	3.1 (2.2)	2.5 (1.5)	0.1 (0.4)	0.0 (0.2)	<0.001
Ranibizumab					
50–59 years	4.3 (4.9)	2.8 (1.7)	0.7 (1.4)	0.5 (1.5)	<0.001
60–69 years	6.8 (7.1)	3.5 (2.0)	1.1 (1.9)	0.9 (1.8)	<0.001
70–79 years	7.2 (7.4)	3.7 (2.1)	1.3 (2.0)	1.0 (2.0)	<0.001
>79 years	5.8 (5.4)	3.5 (1.9)	0.9 (1.7)	0.7 (1.4)	<0.001
All drugs					
50–59 years	4.3 (4.8)	2.9 (1.7)	0.7 (1.4)	0.6 (1.5)	<0.001
60–69 years	6.7 (6.8)	3.6 (2.0)	1.2 (1.9)	0.9 (1.8)	<0.001
70–79 years	6.9 (6.9)	3.8 (2.1)	1.3 (2.0)	1.0 (1.9)	<0.001
>79 years	5.7 (5.0)	3.6 (1.9)	0.9 (1.6)	0.6 (1.4)	<0.001

## Data Availability

All data used for the current study are available upon reasonable request to CliCon S.r.l. Società Benefit, which is the body entitled to data treatment and analysis by Local Health Units.

## Supplementary Materials

The following supporting information can be downloaded at: https://www.mdpi.com/article/10.3390/ijerph19052548/s1, Table S1: Mean number of annual anti-VEGF prescriptions/patient during the follow-up among the wAMD-cohort; Table S2: Mean number of annual anti-VEGF prescriptions/patient during the follow-up among other ocular diseases-cohort patients.
