# Supplementary material for: Retrospective Analysis of the Pharmaco-Utilization of VEGF Inhibitors and Health Care Costs among Patients with Wet Age-Related Macular Degeneration and Other Ocular Diseases in Italy
Source: Int J Environ Res Public Health. 2022 Feb 23;19(5):2548. doi: 10.3390/ijerph19052548 (PMC8909764; doi:10.3390/ijerph19052548)
Supplement: Supplementary file 1 [file ijerph-19-02548-s001.zip › ijerph-1432693-SI.pdf]

Supplementary Material

Table S1. Mean number of annual anti-VEGF prescriptions/patient during the follow-up among the wAMD-cohort.

	All Available FW-UP	1st Year FW-UP	2nd Year FW-UP	3rd Year FW-UP	*P*-Value
N of Treated Patients	2943	3337	2767	2191	
Aflibercept, Mean (SD)	5.6 (3.5)	4.1 (1.8)	1.1 (1.7)	0.5 (1.3)	< 0.001
Pegaptanib, Mean (SD)	4.5 (4.1)	2.7 (1.6)	0.4 (1.1)	0.3 (0.8)	< 0.001
Ranibizumab, Mean (SD)	6.4 (6.6)	3.5 (2.0)	1.1 (1.8)	0.9 (1.7)	< 0.001
Total, Mean (SD)	6.2 (6.2)	3.6 (2.0)	1.1 (1.8)	0.8 (1.7)	< 0.001

Table S2. Mean number of annual anti-VEGF prescriptions/patient during the follow-up among other ocular diseases-cohort patients.

	All Available FW-UP	1st Year FW-UP	2nd Year FW-UP	3rd Year FW-UP	*P*-Value
N of Treated Patients	1648	1877	1564	1215	
Aflibercept, Mean (SD)	4.7 (3.1)	3.7 (1.8)	0.9 (1.5)	0.6 (1.2)	< 0.001
Pegaptanib, Mean (SD)	3.6 (2.3)	2.2 (1.4)	0.7 (1.1)	0.0 (0.0)	< 0.001
Ranibizumab, Mean (SD)	5.0 (4.3)	3.3 (2.0)	0.7 (1.5)	0.5 (1.3)	< 0.001
Total, Mean (SD)	4.9 (4.2)	3.3 (1.9)	0.8 (1.5)	0.5 (1.3)	< 0.001
